# Decellularization Following Fixation of Explanted Aortic Valves as a Strategy for Preserving Native Mechanical Properties and Function

**DOI:** 10.3389/fbioe.2021.803183

**Published:** 2022-01-06

**Authors:** Manisha Singh, Clara Park, Ellen T. Roche

**Affiliations:** ^1^ Institute for Medical Engineering and Science, Massachusetts Institute of Technology, Cambridge, MA, United States; ^2^ Department of Mechanical Engineering, Massachusetts Institute of Technology, Cambridge, MA, United States

**Keywords:** decellularization, aortic valve, hydrodynamic testing, valvular, mechanical properties, aldehyde fixation

## Abstract

Mechanical or biological aortic valves are incorporated in physical cardiac simulators for surgical training, educational purposes, and device testing. They suffer from limitations including either a lack of anatomical and biomechanical accuracy or a short lifespan, hence limiting the authentic hands-on learning experience. Medical schools utilize hearts from human cadavers for teaching and research, but these formaldehyde-fixed aortic valves contort and stiffen relative to native valves. Here, we compare a panel of different chemical treatment methods on explanted porcine aortic valves and evaluate the microscopic and macroscopic features of each treatment with a primary focus on mechanical function. A surfactant-based decellularization method after formaldehyde fixation is shown to have mechanical properties close to those of the native aortic valve. Valves treated in this method were integrated into a custom-built left heart cardiac simulator to test their hemodynamic performance. This decellularization, post-fixation technique produced aortic valves which have ultimate stress and elastic modulus in the range of the native leaflets. Decellularization of fixed valves reduced the valvular regurgitation by 60% compared to formaldehyde-fixed valves. This fixation method has implications for scenarios where the dynamic function of preserved valves is required, such as in surgical trainers or device test rigs.

## Introduction

Cardiovascular benchtop models with mock circulatory loops are increasingly used for surgical training (Ramphal et al., 2005; Mchale and Frank, 2017; Feins et al., 2017), as educational platforms (Gehron et al., 2019; Villanueva et al., 2020), and for research purposes (Chinchoy et al., 2000; Maglio et al., 2021). These systems are typically composed of a mock circulatory loop connected to a pulsatile pump, but often lack anatomical representations and mechanical properties of the components of the heart (Wu et al., 1996; Timms et al., 2005; Taylor and Miller, 2012). Some cardiac simulators utilize *ex vivo* heart tissue to realistically recapitulate the anatomy and flow response (Vismara et al., 2016; Leopaldi et al., 2012), but the shelf-life of freshly explanted tissue without any chemical preservation or perfusion is limited to approximately 4 h. A new class of *in vitro* cardiac simulators use chemically preserved explanted heart tissue to maintain anatomic fidelity (Park et al., 2020), but competent valves are required for utility as *in vitro* cardiovascular simulators for education and training. Therefore, we screened methods that would preserve valves, and ultimately surrounding intracardiac tissue, while ensuring their competency to enable physiological biomechanics and hemodynamics. Hence, there is an unmet need for tissue engineering techniques or chemical treatment methods for native tissues in order to; 1) extend their post-explant longevity, 2) enable practical use in mock-circulatory benchtop models, 3) retain biomechanical properties.

Tissue engineering techniques, such as decellularization via surfactants, can successfully produce tissue scaffolds with mechanical properties similar to native valves, but the resulting scaffolds require special storage conditions (e.g., 4°C, or slow cooling medium, etc.) for their long-term usage and may not be suitable for benchtop applications (Maghsoudlou et al., 2013; Urbani et al., 2017; Gupta et al., 2017). Chemically-induced crosslinking (e.g., through aldehydes or carbodiimides) subsequent to surfactant processing has also been reported to enhance the life-span and storage of the decellularized tissues (Liu et al., 2019) at room temperature. Although thin valve leaflets can easily be decellularized and crosslinked with immersion techniques, successful decellularization of a whole heart requires complex perfusion bioreactors (Guyette et al., 2014).

Human cadaveric heart specimens, which are often chemically treated with formaldehyde, are commonly available in medical schools but stiffen due to crosslinking and are rendered immobile, limiting their use to demonstrating gross heart anatomy and preventing them from being reanimated in mock loops. Chemical preservation of valves utilizes aldehyde-based fixatives which alter the mechanical properties of aortic valve leaflets, hence affecting the function of the valves, and thus the biomimicry of any simulator in which they are installed (Christie, 1992). Glutaraldehyde-fixed leaflets have been reported to be about 100 times stiffer than native leaflets (Talman and Boughner, 1995). Therefore, we screened methods that would restore the mechanical properties of previously preserved valves, and ultimately surrounding intracardiac tissue, while ensuring their competency to enable physiological biomechanics and hemodynamics.

In this study, we compare fixation via formaldehyde and decellularization via surfactants. We limit the scope to aortic valve leaflets and specifically examine treatment effects on the multiscale structure and mechanical properties. We hypothesize that a combination of aldehyde-fixation followed by surfactant-based decellularization may preserve the mechanical properties of native aortic leaflets for use in high fidelity mock circulatory loops and cardiac simulators. For this work, formaldehyde crosslinking has been chosen over glutaraldehyde owing to 1) faster crosslinking, 2) availability of formaldehyde-fixed cadaveric hearts in hospitals and medical schools, and 3) fewer concerns of formaldehyde induced toxicity for *in vitro* applications. Many studies have investigated surfactant-based decellularization involving ionic (e.g., sodium dodecyl sulfate, sodium deoxycholate), non-ionic (e.g., Triton X-100), and a combination thereof (Luo et al., 2019; Zhou et al., 2010). For our purposes, we selected a combination of ionic sodium deoxycholate (SD) and a non-ionic surfactant (Triton X-100) to restore the mechanical function of fixed valves due to superior efficiency in cell removal and matrix preservation over other techniques (Kasimir et al., 2003; Luo et al., 2019). Herein, we assess the impact of SD/Triton X-100 decellularization on formaldehyde-fixed valvular leaflets and compare the mechanical properties of the resulting leaflets with their native counterpart. Furthermore, the effects of surfactants on the micromechanics and morphology of fixed valves were evaluated with Fourier transform infrared spectroscopy (FTIR) and scanning electron microscopy (SEM) and compared with native porcine valves. Next, a physiologically relevant left heart cardiac simulator was custom-built to house the chemically treated aortic valves and their hemodynamic performance was compared to native, fixed, and decellularized valves. At present, cardiac hemodynamics and biomechanics are mainly taught using textbooks and computational models (Mittal et al., 2016). Here, we identify a process that may enable realistic recapitulation of valve function on the bench which enables hands-on cardiac biomechanics education. These chemical treatment techniques have the potential to preserve biomimicry and biomechanics of aortic leaflets while extending their shelf-life so they can be installed in cardiac simulators as an educational or research tool.

## Materials and Methods

### Treatment of Porcine Aortic Valves

Fresh porcine hearts with intact ascending aorta were purchased from Sierra for Medical Science Inc., (California, United States). A 3 cm long segment of the aorta with valves attached at the annulus was dissected from the hearts. Before processing, valves were thoroughly washed with 1X phosphate-buffered saline (PBS) of pH 7.4 to remove all blood and blood clots. For aldehyde-based fixation, the explanted aortic valves (*n* = 3) were submerged in 200 ml of 10% Neutral-buffered Formalin (Sigma Aldrich) over a gentle shaker for 18 h at 25°C and thoroughly washed in 1X PBS thereafter. For decellularization, the explanted valves (*n* = 3) were treated in a 200 ml mixture of 5% (or 10% for increased surfactant concentration) w/v Sodium Deoxycholate (Sigma Aldrich) and 5% (or 10% for increased surfactant concentration) w/v Triton X-100 (Thermo Fisher Scientific) in 1X PBS over gentle shaking for a total of 48 h at 25°C. The solution was refreshed after 24 h during decellularization. For the combination of fixation and decellularization, the valves were first fixed in formaldehyde followed by the surfactant-based decellularization as mentioned above. The fresh, formaldehyde-treated, decellularized aortic valves, and those that were decellularized after fixation are referred to as “native”, “fixed”, “decel”, and “fixed-decel” respectively throughout the text. Two different concentrations of 5 and 10 w/v% of the surfactants were used and referred to as “fixed-decel 5%” and “fixed-decel 10%”, respectively.

### Fourier Transform Infrared Spectroscopy

Dried fresh (*n* = 3) and chemically-treated porcine aortic valves (*n* = 3) were studied using infrared spectroscopy in Attenuated Total Reflection mode using a Thermo Fisher FTIR6700 Fourier Transform Infrared Spectrometer. Data were acquired at a resolution of 4 cm^−1^ with 64 scans and a scan range of 4,000–500 cm^−1^.

### Scanning Electron Microscopy

Fresh (*n* = 3) and chemically treated valves (*n* = 3) were immersed in 2.5% glutaraldehyde for 1 h, dehydrated with ethanol, and then dried for 12 h before embedding in paraffin wax for cross-sectional evaluation. Prior to imaging with a scanning electron microscope, the samples were sputter-coated (50 nm) with gold. Images were acquired by Zeiss Merlin high-resolution electron microscope at an acceleration voltage of 1 kV and a working distance of 4–6 mm.

### Mechanical Performance

The valve leaflets were cut from fresh and chemically treated aortic segments. The leaflets of each type of valve (*n* = 3) were stretched uniaxially in the radial direction at a rate of 10 mm min^−1^ using an Instron 5944 mechanical tester equipped with a 2-kN load cell. Prior to testing, the thickness and size of each leaflet were measured using a micrometer and vernier caliper respectively to calculate stress and strain from the recorded force vs. displacement data. From literature, it is evident that the strength of an aortic valve leaflet is weaker in the radial direction than circumferential. Testing in the weaker (i.e., radial) direction was performed as it represented the worst case condition for cyclic hydrodynamic testing.

### Hydrodynamic Assessment

A mock circulatory loop representing the left heart circulation was built in-house for hydrodynamic evaluation of the aortic valves. A pulsatile pump (Harvard Apparatus, United States) was connected to the loop to create a pulsatile flow at 60 beats per min under physiological conditions at a cardiac output of 3.6 L min^−1^. The aortic valve was sutured on a sewing ring on a 3D-printed adaptor and mounted onto the circuit. Hydrodynamic parameters were measured in real-time using pressure sensors (PendoTECH) before and after the valve and an ultrasonic flow probe (Transonic) placed after the valve and connected to a T420 multi-channel research console (Transonic). An endoscopic camera (NIDAGE wireless) was used to record real-time valve function and leaflet motion. Sensor output was acquired for approximately 10 beats per sample using PowerLab (ADInstruments). A total of three samples were used for each valve type except for the mechanical valve. Only one specimen was used for mechanical valve due to its high repeatability and robustness.

### Statistical Analysis

All data are presented as mean ± Std Dev, with *n* = 3, unless stated otherwise. The *p*-values (*p < 0.05) are calculated using one-way ANOVA with Tukey correction in Origin Pro 2019 (64-bit) and ns denotes no statistical difference.

## Results

### Micromechanics and Surface Characterization of Valve Leaflets

**FIGURE 1:**
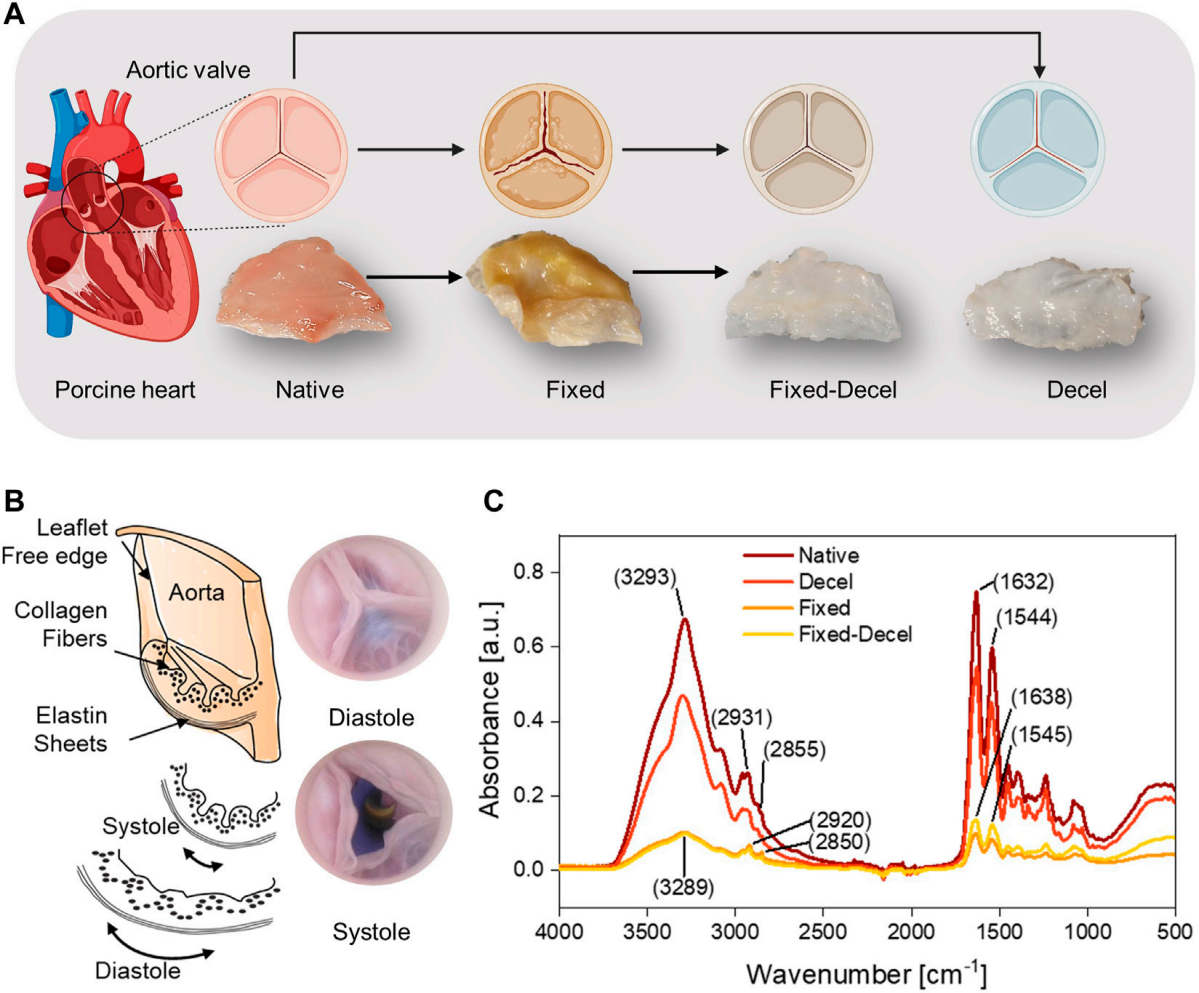
Overall scheme and study design. **(A)** Porcine aortic valves are explanted and either chemically crosslinked with fixative formaldehyde (fixed), or decellularized with surfactants Triton X-100/sodium deoxycholate (decel) or treated with a combination of fixative and surfactants (fixed-decel) to optimize the biomechanical properties. Digital photos of the cut leaflets show that decel and fixed-decel groups were discolored due to the removal of cell and related membrane proteins by the surfactants. Fixed leaflets appear to be distorted in shape relative to the native valve and the original shape was recovered when fixed leaflets were decellularized (fixed-decel). **(B)** Digital pictures and a cross-section schematic of an aortic valve showing composition and orientation of collagen and elastin fibers in different layers during systole and diastole. **(C)** Fourier Transform Infrared Spectroscopy (FTIR) spectra indicate the peak intensity and location of characteristic chemical groups.

Aortic valves function in a mechanically demanding environment that requires micromechanical remodeling of the leaflet tissue. Explanted valves require chemical treatments to match the inherent mechanical properties of the native valvular tissue. Among various chemical treatments, aldehyde-based fixation is considered the gold standard but causes the aortic valves to stiffen and mechanically deform (Figure 1A). Decellularization is an alternative technique but results in a weak scaffold that is prone to material failure. In this study, we have adopted a combination of aldehyde-based fixation and surfactant-based decellularization to yield mechanically strong yet soft leaflets that mimic the native valves closely (Figure 1A). The native leaflets are thin, flexible, and mainly comprised of collagen and elastin fibers. The collagen fibers are corrugated and arranged in the circumferential direction whereas elastin fibers are positioned in the radial direction (Figure 1B). During diastole, continuous stretching of elastin occurs which causes the initially crimped collagen fibers to uncrimp from a wavy configuration to straight. To detect any conformational and structural alterations in the collagen and elastin fibers due to various chemical treatments, Fourier Transform Infrared Spectroscopy (FTIR) was performed. The overlay of FTIR spectra of treated and control samples showed no new absorption peaks but detected changes in peak intensity and positions (Figure 1C). The wide band from 3,500 to 3,200 cm^−1^ corresponds to the symmetric and asymmetric stretching vibrations of -OH and -NH functional groups. The wide peak intensity was reduced by 85% for fixed samples, indicating covalent interactions of amine and hydroxyl groups through aldehyde crosslinking. The asymmetric and symmetric -CH_2_ stretching bands from elastin and collagen structures are located at 2,950–2,850 cm^−1^. The absence of an aldehyde characteristic peak in fixed and fixed-decel samples at 1730 cm^−1^ indicates that no free aldehyde groups are present, and leaflets have been washed thoroughly. The 40% increase in the intensity of amide I (−CO-NH− I) and amide II (−CO-NH− II) peaks at 1,630 cm^−1^ and 1,550 cm^−1^ for fixed-decel samples indicates the conformation and orientation changes in the secondary protein’s structure of collagen and elastin as compared to fixed groups.

**FIGURE 2:**
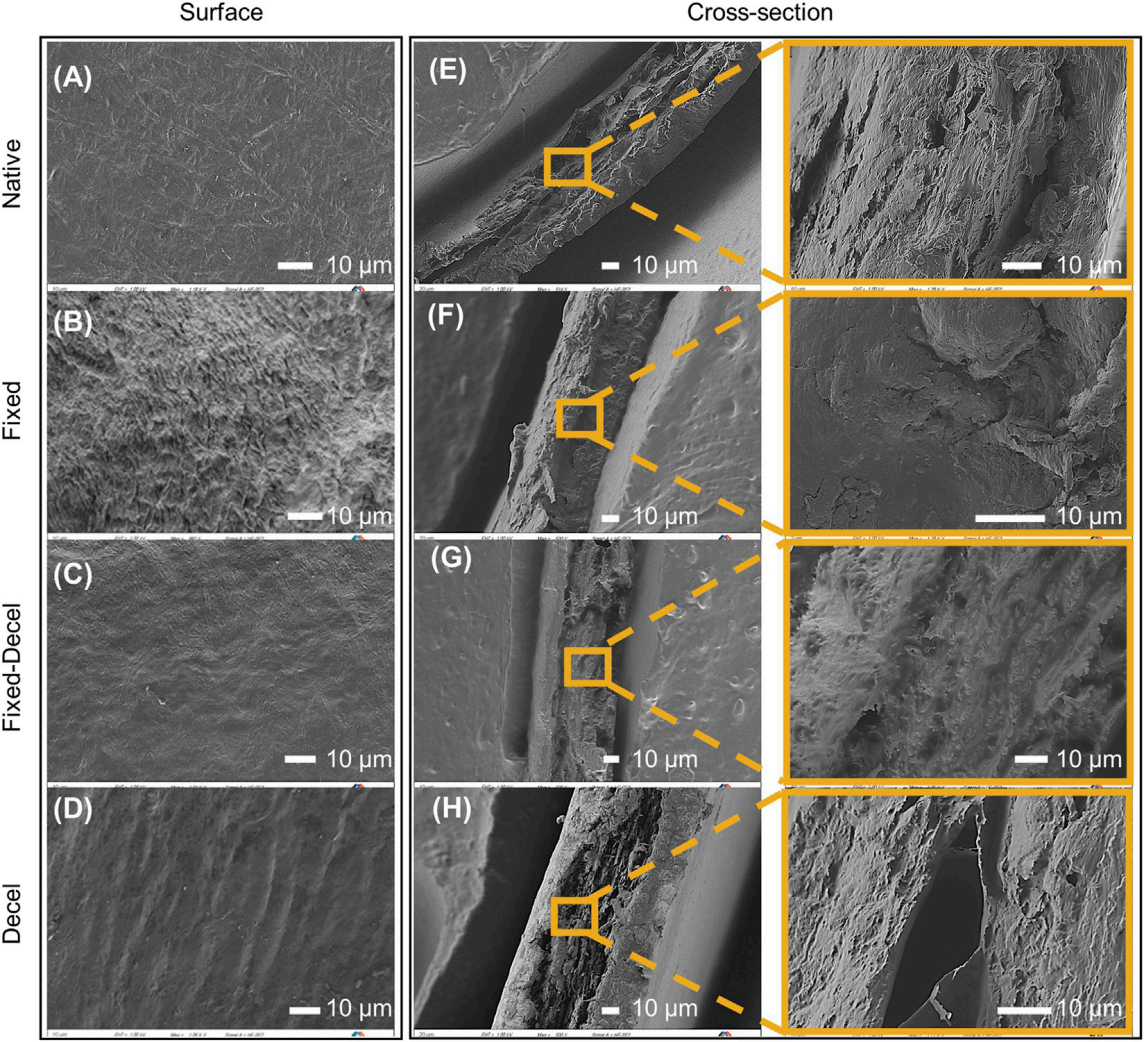
Surface characterization using scanning electron microscopy (SEM). **(A–D)** SEM micrographs showing the morphology of leaflets from the surface. **(E–H)** Micrographs showing cross-sectional morphology of leaflets that have been fixed, decellularized, and a combination thereof including the native control. The scale bar is 10 µm for all.

FTIR surface characterization is also supported by the morphology of the valvular leaflets (Figure 2). The crimping of collagen and elastin fibers is observed in the case of fixed samples when imaged under a scanning electron microscope from the surface (Figure 2B). No obvious difference in pore size was observed for native, decel, and fixed-decel groups (Figures 2A,C,D). The decel group showed thinner fiber structures and loose surface texture when compared to native valves (Figure 2D). This is likely due to detergent-mediated solubilization and dissociation of cell membranes. Cross-sectioned morphology exhibits that the fibers in the fixed group are much more tightly packed than the fixed-decel samples (Figures 2F,G). The increased porosity in fixed-decel samples could be attributed to the solubilization of membrane proteins by surfactants. SEM also confirmed that the cells were removed from the decel group, therefore causing the collagen fibers to loosen to a porous structure (Figure 2H).

### Combination of Fixation and Decellularization Yields Native Tissue-Mimicking Mechanical Properties

**FIGURE 3:**
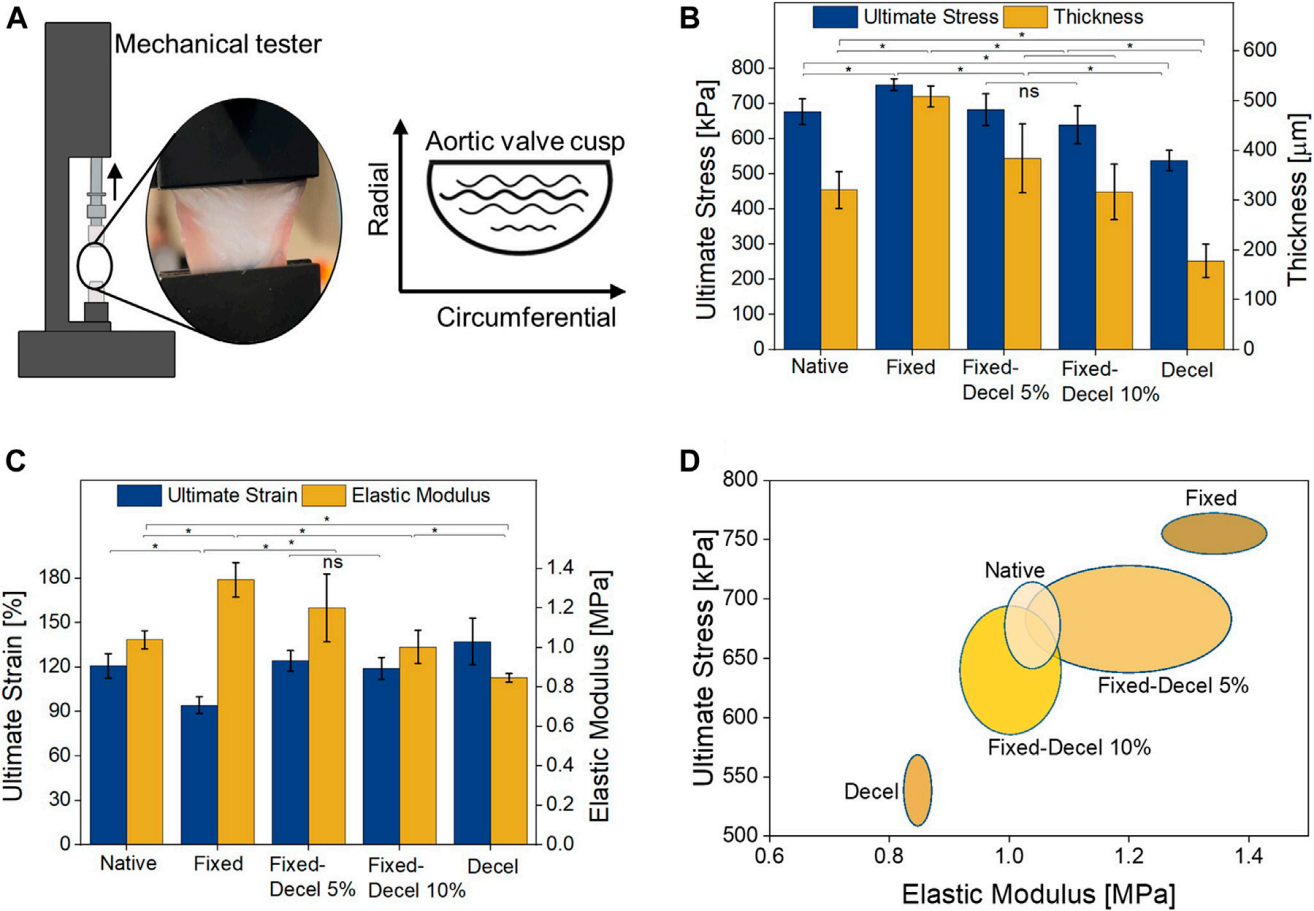
Evaluation of mechanical properties. **(A)** Set up of uniaxial tensile test performed in the radial direction. **(B)** Comparison of leaflet thickness and maximum stress at failure **(C)** Comparison of elastic modulus (i.e., stiffness) and maximum strain at failure. **(D)** two-dimensional comparison (Elastic modulus vs. ultimate stress at break) of mechanical properties among fixed, decellularized, and a combination thereof, including the native control groups. Data presented as mean ± Std. Dev., *n* = 3, *p*-values are calculated using one-way ANOVA with Tukey correction, *p < 0.05 in Origin Pro 2019 (64-bit); ns denotes no statistical difference).

Due to the circumferential orientation of collagen fibers, leaflets are stiffer in the circumferential direction than the radial while exhibiting more extensibility in the radial direction. To compare the mechanical properties of chemically-treated leaflets with native, a uniaxial tensile test was performed in the radial direction (Figure 3A). Prior to mounting the samples in a mechanical tester, the thickness of each leaflet was measured with a digital micrometer. The thickness of fixed specimens increased by 67% whereas it decreased by 45% for decellularized samples when compared to native aortic valves (Figure 3B). The combination of fixed and decellularization resulted in a thickness comparable to the native tissue. As the weight of surfactants for decellularization was increased from 5 to 10%, a further reduction of 16% was noted in the mean value of thickness for fixed-decel groups, although it is not statistically significant according to one-way ANOVA with Tukey correction. Fixed samples have the highest ultimate stress of 755 ± 17 kPa which is significantly higher than the native porcine aortic valves 677 ± 36 kPa (Figure 3B). Combination of fixation and decellularization yielded ultimate stress of 683 ± 44 kPa and 640 ± 54 kPa for 5 and 10% surfactant concentration, respectively. Decellularization alone reduced the maximum stress at the failure to 538 ± 29 kPa, which is significantly weaker than native leaflets. Similar trends were observed in the modulus of elasticity (calculated under 5% strain levels) where fixation alone resulted in significantly stiffer (1.4 ± 0.09 MPa) samples as compared to native tissue (1 ± 0.05 MPa) (Figure 3C). Fixed-decel groups yielded an elastic modulus of 1.2 ± 0.2 MPa and 1 ± 0.08 MPa for 5 and 10% surfactant concentration respectively, both of which matched closely (*p* > 0.05; one-way ANOVA with Tukey correction) with native tissues. Decellularized groups exhibited the lowest modulus of elasticity 0.8 ± 0.02 MPa. There is no statistically significant difference between the ultimate strain of native aortic valves 120 ± 8% to that of fixed-decel 124 ± 6% and 119 ± 7% for 5 and 10%, respectively (Figure 3C). Fixed samples exhibited ultimate tensile strains of 94 ± 5%, which is 1.3 times lower than the native group. Ultimate stress and elastic modulus are plotted for a two-dimensional comparison of chemically treated groups with native control (Figure 3D). An overlap in the mechanical properties was observed for fixed-decel samples whereas the pristine decel and fixed samples showed a contrasting difference in comparison to native leaflets. From the data above, it can be concluded that the combination of fixation and decellularization techniques matches the mechanical properties of native aortic tissues whereas the controls of fixation and decellularization alone are either too stiff or weak.

### Hydrodynamic Assessment of Chemically Treated Aortic Valves

**FIGURE 4:**
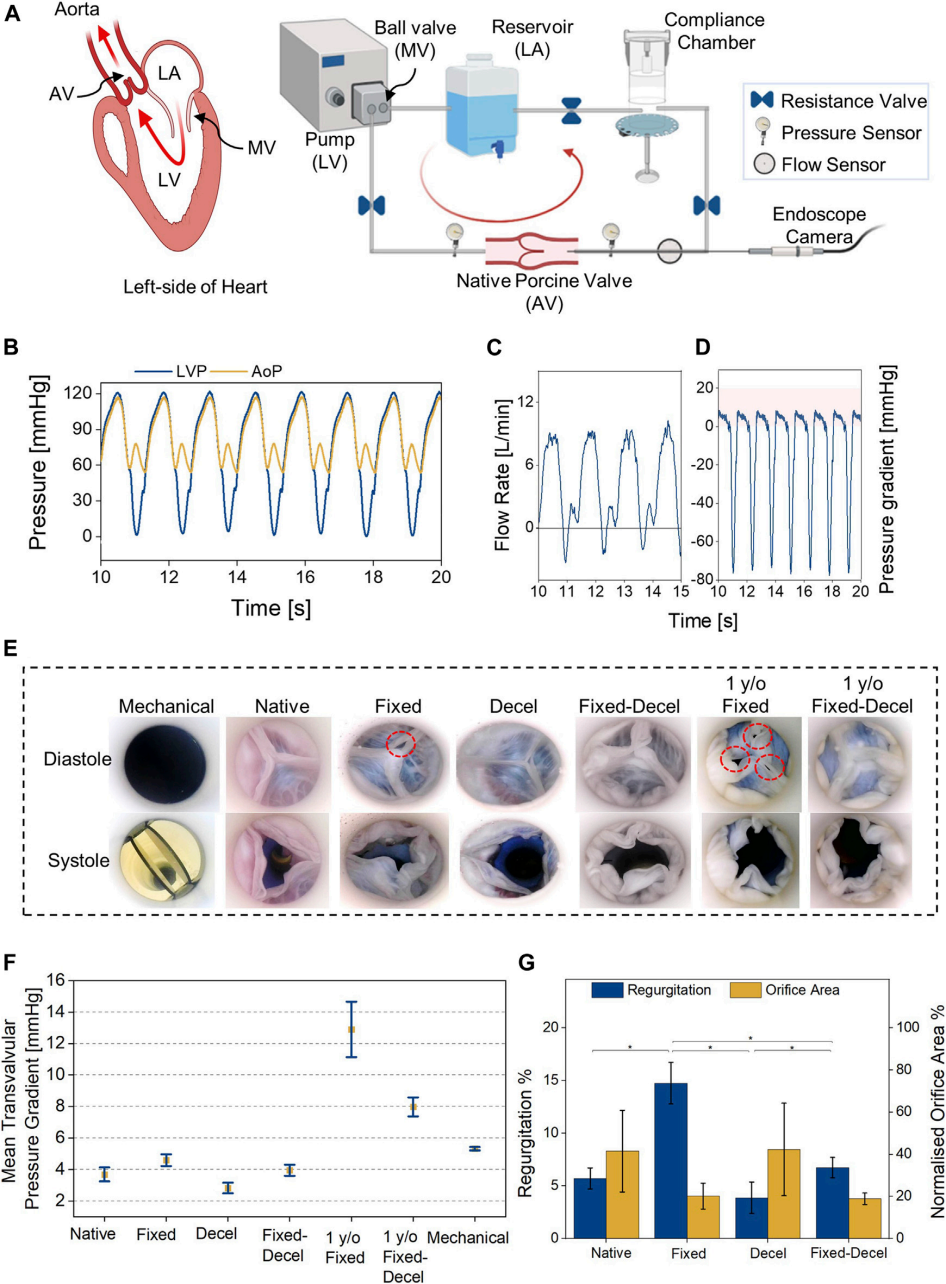
Hydrodynamic assessment. **(A)** Custom-built benchtop setup with a pulsatile mock circulatory loop representing left heart circulation. **(B,C)** Representative pressure and flow waveforms for native aortic valves at 60 bpm and cardiac output of 3.6 L/min. **(D)** Representative transvalvular pressure gradients for native valves over a few cardiac cycles. **(E)** Digital images were captured using an endoscope camera for different groups of aortic valves at peak systole and diastole. **(F)** Comparison of mean transvalvular pressure gradients (ΔP) among fixed, decellularized, and a combination thereof, including the native and commercially available mechanical (St. Jude) control groups. **(G)** Comparison of regurgitant flow and opening orifice area at peak systole. Data presented as mean ± Std Dev, *p*-values are calculated using one-way ANOVA with Tukey correction, *p < 0.05 in Origin Pro 2019 (64-bit), ns denotes no statistical difference. LA = left atrium, LV = left ventricle, MV = mitral valve. Decel = decellularized.

For evaluating the hydrodynamic performance of chemically treated aortic valves, we built a benchtop setup with a pulsatile mock circulatory loop mimicking the left heart circulation (Figure 4A). Along with the anatomically accurate aortic valves, our custom-built flow setup houses a reservoir to store the fluid, a compliance chamber to mimic the aortic compliance, and a pulsatile pump to displace fluid at physiologically relevant ventricular stroke volumes (SV) and heart rates. Left ventricular (0–120 mmHg) (LVP) and aortic (60–120 mmHg) pressure (AoP) conditions are simulated by controlling the resistance valves in the flow circuit. Two pressure sensors were placed before and after the valves to acquire the real-time pressures during valve performance testing. A flow probe was placed downstream of the aortic valve to record any changes in the forward and backward flow. The recorded left ventricle and aortic pressures and flow for native porcine valves at 60 bpm and SV of 60 ml over a few cardiac cycles are shown in Figures 4B,C. Based on LVP and AoP readings, the mean transvalvular systolic pressure gradients for native porcine valves were calculated and plotted in Figure 4D. Aortic flow data was used to calculate the regurgitation %, which represents the ratio of the regurgitant volume and the stroke volume, expressed in percentage. An endoscopic camera records the real-time leaflet motion during systole and diastole phases (Figure 4E, Supplementary Video S1). Normalized orifice area determines the ratio of the geometrical opening area of the valves at peak systole and the cross-sectional area of the attached aorta. The geometrical opening area of the valves was calculated using ImageJ (Abràmoff et al., 2004) from the recorded endoscope camera videos. The representative images in Figure 4E and Supplementary Video S1 qualitatively compare the opening (during systole) and closing (during diastole) of chemically treated valves relative to the native/fresh aortic valve and a mechanical St. Jude valve. The aldehyde-based crosslinking continues over time, and treated tissues turn stiffer with age (Sacks and Liao, 2018). In order to account for the stiffening of fixed valves with time, we included an additional control group comprising of one-year-old fixed aortic valves. As seen in Figure 4E, the fixed valves turn stiff and do not completely close at diastole. The mechanically deformed area related to the partial closure is highlighted by red dotted circles, which is more apparent in the one-year-old samples. When the fixation was combined with decellularization for the same valves, no gaps were visible between the valve leaflets during diastole (Figure 4E). Mean transvalvular pressure gradients (ΔP) also support this observation and ΔP required for complete closure are significantly different in the case of chemically-treated valves relative to the native (Figure 4F). The recorded ΔP for fixed and decel groups are 4.6 ± 0.4 and 2.8 ± 0.3 mmHg, respectively which are statistically different (*p* < 0.05; one-way ANOVA with Tukey correction) when compared to native 3.7 ± 0.4 mmHg and fixed-decel 3.9 ± 0.3 mmHg groups. One-year-old (y/o) formaldehyde-fixed groups exhibited an ΔP of 13.0 ± 1.8 mmHg, which was reduced to 8.0 ± 0.6 mmHg when followed by decellularization. Fixation after decellularization clearly reduced the transvalvular pressure gradients relative to fixed groups, but no significant differences (*p* > 0.5) were noted in the normalized orifice area opening at peak systole of fixed vs. fixed-decel groups (Figure 4G). However, owing to the synergistic effect of reduced stiffness and ultimate strain (Figure 3C) for fixed-decel versus fixed groups, complete closure of fixed-decel groups was observed at peak diastole relative to fixed groups (Figure 4E). This observation is also in line with the regurgitant flow calculations (Figure 4G). The fixed groups showed the highest regurgitation of 15 ± 2% which was lowered to 6 ± 0.9% for fixed-decel groups that match closely with native 5.7 ± 1% groups (Figure 4G).

## Discussion

In this work, we have demonstrated that hemodynamic function and biomechanical properties of formaldehyde-fixed aortic valve tissues can be improved when treated with a combination of sodium deoxycholate (SD)/Triton X-100 surfactants. We have compared several chemical treatment methods on explanted porcine aortic valves and evaluated microscopic and macroscopic features of each treatment with a primary focus on mechanical function. Our results show that the decellularization process lowers the ultimate strength and elastic modulus of aldehyde-fixed valve leaflets. This reduction in mechanical strength (i.e., ultimate stress and elastic modulus) of the fixed tissues may be attributed to 1) modification of structural components, such as collagen and/or elastin fiber rearrangements (Figure 2), 2) solubilization and dissociation of cell membranes by the detergents (Figure 1C), or 3) a combination thereof.

The resulting valves with biomimicking mechanical properties can be directly employed in cardiac simulators for educational and training purposes, as shown in our left heart pulsatile flow loop equipped with real-time hydrodynamic readings and intravascular imaging. Using this technique to restore the mechanics of preserved tissues can potentially be extended to high-fidelity physical heart simulators with utility in medical education or procedural planning. For instance, human cadaveric heart specimens, which are commonly used in anatomy labs in medical schools to teach gross anatomy (James et al., 2019), may be restored to have competent valve function and attached to a mock circulatory loop. Further, the preserved hearts can potentially be reanimated using a biohybrid approach (Park et al., 2020) to produce an interactive, beating heart model which would complement the current educational tools with an enhanced learning and training experience. In the future, this technique can potentially be employed for building accurate *in vitro* prototypes to experimentally validate computational models, such as fluid-structure interaction with dynamic function of the aortic valve (Dumont et al., 2004) or for device testing and procedural planning purposes.

Several limitations of the current study should be noted—1) the panel of treatment options did not include decellularization followed by fixation, which is commonly used for bioprosthetic valves made from pericardium for implantation but was excluded from our work as we wish to extend the treatment process to the whole heart, including the vessels and myocardial tissue, ideally without using reperfusion bioreactors for ease of manufacturability; 2) for a proof-of-concept, we selected one protocol for decellularization (i.e., the combination of SD and Triton X-100) and our current study lacks a detailed comparison of the several other alternative decellularization methods described in the literature; 3) since this work focuses on restoring native valve mechanical properties in aldehyde-fixed valves for benchtop applications, the degree of cell removal has not been assessed. Before adaptation of this work for any *in vivo* or tissue engineering application, detailed characterization on cell removal and toxicity is required; 4) preliminary hydrodynamic testing use water which does not mimic the density and viscosity of blood. A monoethylene glycol-based blood-mimicking fluid as described in our previous work (Singh et al., 2021) could improve the hydrodynamic accuracy; 5) since the valves were manually sutured to an adaptor to align them normal to the flow circuit, the natural annulus size and tension applied to the leaflets may have been altered, potentially leading to variability in the normalized orifice area; 6) preliminary hydrodynamic testing does not include the fatigue performance of the valves under pulsatile physiological loading and is part of our future work.

In conclusion, we find that a surfactant-based decellularization technique can restore the native biomechanical properties of aldehyde-fixed aortic valves. The ability to retrospectively decellularize fixed valves and reanimate them in benchtop simulators to recapitulate biomechanics has utility in cardiovascular education, training, and research.

## Data Availability

The original contributions presented in the study are included in the article/Supplementary Material, further inquiries can be directed to the corresponding author.

## References

[B1] Abràmoff M. D. Magalhães P. J. Ram S. J. (2004). Image Processing with ImageJ. Biophotonics Int. 11 (7), 36–42.

[B2] Chinchoy E. Soule C. L. Houlton A. J. G. Gallagher W. J. Hjelle M. A. Laske T. G. (2000). Isolated Four-Chamber Working Swine Heart Model. Ann. Thorac. Surg. 70 (5), 1607–1614. 10.1016/s0003-4975(00)01977-9 11093495

[B3] Christie G. (1992). Anatomy of Aortic Heart Valve Leaflets: the Influence of Glutaraldehyde Fixation on Function. Eur. J. cardio-thoracic Surg. 6 (Suppl. ment_1), S25–S33. 10.1016/1010-7940(92)90018-s 1389275

[B4] Dumont K. Stijnen J. M. A. Vierendeels J. van de Vosse F. N. Verdonck P. R. (2004). Validation of a Fluid-Structure Interaction Model of a Heart Valve Using the Dynamic Mesh Method in Fluent. Comput. Methods Biomech. Biomed. Eng. 7 (3), 139–146. 10.1080/10255840410001715222 15512757

[B5] Feins R. H. Burkhart H. M. Conte J. V. Coore D. N. Jr Fann J. I. Hicks G. L. (2017). Simulation-based Training in Cardiac Surgery. Ann. Thorac. Surg. 103 (1), 312–321. 10.1016/j.athoracsur.2016.06.062 27570162

[B6] Gehron J. Zirbes J. Bongert M. Schäfer S. Fiebich M. Krombach G. (2019). Development and Validation of a Life-Sized Mock Circulatory Loop of the Human Circulation for Fluid-Mechanical Studies. ASAIO J. 65 (8), 788–797. 10.1097/mat.0000000000000880 30281544

[B7] Gupta S. K. Mishra N. C. Dhasmana A. (2017). “Decellularization Methods for Scaffold Fabrication,” in Decellularized Scaffolds and Organogenesis (Berlin, Germany: Springer), 1–10. 10.1007/7651_2017_34

[B8] Guyette J. P. Gilpin S. E. Charest J. M. Tapias L. F. Ren X. Ott H. C. (2014). Perfusion Decellularization of Whole Organs. Nat. Protoc. 9 (6), 1451–1468. 10.1038/nprot.2014.097 24874812

[B9] James H. K. Chapman A. W. Pattison G. T. R. Griffin D. R. Fisher J. D. (2019). Systematic Review of the Current Status of Cadaveric Simulation for Surgical Training. Br. J. Surg. 106 (13), 1726–1734. 10.1002/bjs.11325 31573088 PMC6900127

[B10] Kasimir M.-T. Rieder E. Seebacher G. Silberhumer G. Wolner E. Weigel G. (2003). Comparison of Different Decellularization Procedures of Porcine Heart Valves. Int. J. Artif. Organs 26 (5), 421–427. 10.1177/039139880302600508 12828309

[B11] Leopaldi A. M. Vismara R. Lemma M. Valerio L. Cervo M. Mangini A. (2012). *In Vitro* hemodynamics and Valve Imaging in Passive Beating Hearts. J. Biomech. 45 (7), 1133–1139. 10.1016/j.jbiomech.2012.02.007 22387122

[B12] Liu J. Jing H. Qin Y. Li B. Sun Z. Kong D. (2019). Nonglutaraldehyde Fixation for off the Shelf Decellularized Bovine Pericardium in Anticalcification Cardiac Valve Applications. ACS Biomater. Sci. Eng. 5 (3), 1452–1461. 10.1021/acsbiomaterials.8b01311 33405620

[B13] Luo Y. Lou D. Ma L. Gao C. (2019). Optimizing Detergent Concentration and Processing Time to Balance the Decellularization Efficiency and Properties of Bioprosthetic Heart Valves. J. Biomed. Mater. Res. 107 (10), 2235–2243. 10.1002/jbm.a.36732 31125175

[B14] Maghsoudlou P. Georgiades F. Tyraskis A. Totonelli G. Loukogeorgakis S. P. Orlando G. (2013). Preservation of Micro-architecture and Angiogenic Potential in a Pulmonary Acellular Matrix Obtained Using Intermittent Intra-tracheal Flow of Detergent Enzymatic Treatment. Biomaterials 34 (28), 6638–6648. 10.1016/j.biomaterials.2013.05.015 23727263 PMC3988964

[B15] Maglio S. Park C. Tognarelli S. Menciassi A. Roche E. T. (2021). High Fidelity Physical Organ Simulators: from Artificial to Bio Hybrid Solutions. IEEE Trans. Med. Robotics Bionics 3, 349. 10.1109/tmrb.2021.3063808

[B16] Mchale J. Frank F. (2017). Beating Heart Controller and Simulator. Google Patents.

[B17] Mittal R. SeoSeo J. H. Vedula V. ChoiChoi Y. J. Liu H. Huang H. H. (2016). Computational Modeling of Cardiac Hemodynamics: Current Status and Future Outlook. J. Comput. Phys. 305, 1065–1082. 10.1016/j.jcp.2015.11.022

[B18] Park C. Fan Y. Hager G. Yuk H. Singh M. Rojas A. (2020). An Organosynthetic Dynamic Heart Model with Enhanced Biomimicry Guided by Cardiac Diffusion Tensor Imaging. Sci. Robot 5 (38), 9106. 10.1126/scirobotics.aay9106 PMC7545316 33022595

[B19] Ramphal P. S. Coore D. N. Craven M. P. Forbes N. F. Newman S. M. Coye A. A. (2005). A High Fidelity Tissue-Based Cardiac Surgical Simulator. Eur. J. Cardio-Thoracic Surg. 27 (5), 910–916. 10.1016/j.ejcts.2004.12.049 15848335

[B20] Sacks M. S. Liao J. (2018). Advances in Heart Valve Biomechanics: Valvular Physiology, Mechanobiology, and Bioengineering. Berlin, Germany: Springer.

[B21] Singh M. Varela C. E. Whyte W. Horvath M. A. Nigel C. S. T. Ong C. B. (2021). Minimally Invasive Electroceutical Catheter for Endoluminal Defect Sealing. Sci. Adv. 7 (14), eabf6855. 10.1126/sciadv.abf6855 33811080 PMC11057783

[B22] Talman E. A. Boughner D. R. (1995). Glutaraldehyde Fixation Alters the Internal Shear Properties of Porcine Aortic Heart Valve Tissue. Ann. Thorac. Surg. 60 (2 Suppl. l), S369–S373. 10.1016/0003-4975(95)00250-o 7646190

[B23] Taylor C. E. Miller G. E. (2012). Mock Circulatory Loop Compliance Chamber Employing a Novel Real-Time Control Process. J. Med. Device 6 (4), 450031–450038. 10.1115/1.4007943 23904906 PMC3707194

[B24] Timms D. Hayne M. McNeil K. Galbraith A. (2005). A Complete Mock Circulation Loop for the Evaluation of Left, Right, and Biventricular Assist Devices. Artif. Organs 29 (7), 564–572. 10.1111/j.1525-1594.2005.29094.x 15982285

[B25] Urbani L. Maghsoudlou P. Milan A. Menikou M. Hagen C. K. Totonelli G. (2017). Long-term Cryopreservation of Decellularised Oesophagi for Tissue Engineering Clinical Application. PloS one 12 (6), e0179341. 10.1371/journal.pone.0179341 28599006 PMC5466304

[B26] Villanueva C. Xiong J. Rajput S. (2020). Simulation‐based Surgical Education in Cardiothoracic Training. ANZ J. Surg. 90 (6), 978–983. 10.1111/ans.15593 31828909

[B27] Vismara R. Leopaldi A. M. Piola M. Asselta C. Lemma M. Antona C. (2016). *In Vitro* assessment of Mitral Valve Function in Cyclically Pressurized Porcine Hearts. Med. Eng. Phys. 38 (4), 346–353. 10.1016/j.medengphy.2016.01.007 26908180

[B28] Wu Z. J. Gao B. Z. Slonin J. H. Hwang N. H. C. (1996). Bileaflet Mechanical Heart Valves at Low Cardiac Output. Asaio J. 42 (5), M747–M749. 10.1097/00002480-199609000-00088 8944981

[B29] Zhou J. Fritze O. Schleicher M. Wendel H.-P. Schenke-Layland K. Harasztosi C. (2010). Impact of Heart Valve Decellularization on 3-D Ultrastructure, Immunogenicity and Thrombogenicity. Biomaterials 31 (9), 2549–2554. 10.1016/j.biomaterials.2009.11.088 20061016

